# Sleep education in pediatric residency programs: a cross-cultural look

**DOI:** 10.1186/1756-0500-6-130

**Published:** 2013-04-03

**Authors:** Jodi A Mindell, Alex Bartle, Youngmin Ahn, Mahesh Babu Ramamurthy, Huynh Thi Duy Huong, Jun Kohyama, Albert M Li, Nichara Ruangdaraganon, Rini Sekartini, Arthur Teng, Daniel YT Goh

**Affiliations:** 1 The Children’s Hospital of Philadelphia and Saint Joseph’s University, Philadelphia, USA; 2 Sleep Well Clinics, Christchurch, New Zealand; 3 Eulji University School of Medicine, Daejeon, South Korea; 4 National University Hospital, Singapore, Singapore; 5 University of Medicine and Pharmacy, HCMC, Ho Chi Minh, Vietnam; 6 Tokyo Bay Urayasu/Ichikawa Medical Center, Tokyo, Japan; 7 Prince of Wales Hospital, The Chinese University of Hong Kong, Ma Liu Shui, Hong Kong; 8 Faculty of Medicine, Mahidol University, Nakhon Pathom, Thailand; 9 Medical School University of Indonesia, Jakarta, Indonesia; 10 Sydney Children’s Hospital and University of New South Wales, Sydney, Australia; 11 National University of Singapore, Singapore, Singapore

**Keywords:** Sleep, Sleep disorders, Residency, Education, Pediatrics

## Abstract

**Background:**

The objective of this study was to assess the prevalence of education about sleep and sleep disorders in pediatric residency programs and to identify barriers to providing such education.

**Methods:**

Surveys were completed by directors of 152 pediatric residency programs across 10 countries (Hong Kong, India, Indonesia, Japan, Singapore, South Korea, Thailand, United States-Canada, and Vietnam).

**Results:**

Overall, the average amount of time spent on sleep education is 4.4 hours (median = 2.0 hours), with 23% responding that their pediatric residency program provides no sleep education. Almost all programs (94.8%) offer less than 10 hours of instruction. The predominant topics covered include sleep-related development, as well as normal sleep, sleep-related breathing disorders, parasomnias, and behavioral insomnia of childhood.

**Conclusions:**

These results indicate that there is still a need for more efforts to include sleep-related education in all pediatric residency programs, as well as coverage of the breadth of sleep-related topics. Such education would be consistent with the increased recognition of the importance of sleep and under-diagnosis of sleep disorders in children and adolescents.

## Background

Sleep disorders are highly prevalent in children and adolescents. Unfortunately they are often unrecognized and underdiagnosed [1], which can significantly impact mortality, morbidity, and quality of life [2]. For example, a recent study of the prevalence of diagnosed sleep disorders in pediatric primary care practices found that only 3.7% of children and adolescents were diagnosed with an ICD-9 sleep disorder, which is significantly lower than prevalence rates in epidemiological studies. One contributing factor for this low rate of recognition is the low awareness stemming from the limited education about sleep and sleep disorders in pediatric education [3]. Core competencies for sleep education were identified back in 2003, but there seems to be continued limited education in this area [4].

The three studies to date that have investigated the inclusion of education about sleep and sleep medicine in medical schools were conducted in 1979, 1990, and most recently in 2011 [5-7]. These studies have found that typically less than 2 hours of sleep education are provided during medical school, with minimal change across the last 30 years. The only study of sleep education for pediatricians found an average of 4 hours of didactic lectures specific to pediatric sleep [8]. Furthermore, surveys of practicing pediatricians find greatly limited sleep knowledge, thus further emphasizing the need for education on sleep and sleep disorders both during medical school and during pediatric residency. One study of pediatricians found limited total knowledge about sleep, with approximately half of respondents reporting that they did not feel confident screening for sleep problems and the majority not feeling confident in their ability to evaluate (66%) or treat (75%) sleep problems in children [9]. A recent study of 346 pediatricians found that 96% believed it was their job to counsel patients/caregivers regarding sleep hygiene, however very few (18%) reported ever receiving formal training on sleep disorders [10]. Clearly there is a great need for advancement of sleep education in pediatrics.

To this end, the objective of this study was to investigate the inclusion of sleep education in pediatric residency programs. Pediatric residency programs were surveyed by members of the Asia-Pacific Pediatric Sleep Alliance (APPSA), a group of pediatric and sleep medicine specialists whose mission is to improve the understanding and management of sleep and sleep-related disorders in children across the Asia-Pacific region. The primary aims were to (1) assess the prevalence of didactic and non-didactic sleep education about sleep and sleep disorders in pediatric residency and (2) identify barriers to providing such education.

## Method

A brief survey was sent to the directors of pediatric residency programs in 10 countries in the Asia-Pacific region (see Additional file 1), including Hong Kong (HK; n = 11), India (IN; n = 38), Indonesia (ID; n = 10), Japan (JP; n = 525), Singapore (SG; n = 2), South Korea (n = 57), Thailand (TH; n = 15), and Vietnam (VN; n = 7). We also surveyed pediatric residency programs in the United States and Canada (US-CA; n = 197) as a comparison.

The survey was based on questionnaires used in previous studies [6,8] with the majority of the survey identical to one that was completed in a previous study of medical schools [7] to enable comparisons across educational modalities. The first section asked about the amount of time allocated to sleep education in 6 domains (cardiology, ENT/otololaryngology, neurology, respiratory/pulmonology, development, and psychiatry/psychology). The second section asked what sleep topics were covered in 7 areas (circadian rhythm disorders, hypersomnia, insomnia, pediatric sleep disorders, parasomnias, sleep apnea, and sleep related movement disorders) for adults and in pediatrics. Questions were asked about non-didactic sleep-related education opportunities, including non-elective and elective rotations, grand round speakers, guest lectures, case consults, and journal clubs. Finally, the respondents were asked to identify if any of the following barriers occurred if sleep was not included in their curriculum (insufficient time, lack of trained staff/qualified instructors, lack of resources, lower priority, or not relevant to the program). In total, the survey included 24 questions and took approximately 3–5 minutes to complete.

This study was approved by the Institutional Review Board at Saint Joseph’s University. All surveys were completed online anonymously and all residency directors were contacted 2 or 3 times.

### Statistical analyses

Descriptive data, including mean, median, and frequencies, are presented for all variables. Analyses of variance were used to compare across countries for all continuous variables. Chi-square analyses were conducted for categorical variables.

## Results

### Response rate

Similar to the previous study of medical schools, one of the most striking findings was the lack of response. Overall, only 17.6% (34.4% excluding Japan) of the residency programs contacted (n = 152 of 865) responded. By country, response rates ranged from 7% to 100%; specifically Hong Kong (27.3%; n = 3 of 11), India (44.7%; n = 17 of 38), Indonesia (90%; n = 9 of 10), Japan (6.7%; n = 35 of 525), Singapore (100%; n = 2 of 2), South Korea (35.1%; n = 20 of 57), Thailand (6.7%; n = 1 of 15), United States-Canada (32.5%; n = 64 of 197), and Vietnam (10.0%; n = 1 of 10).

### Prevalence of sleep education

All results reported are based on completed surveys. As seen in Table 1, across all pediatric residency programs, the sleep-related area that the highest percentage of schools reported covering was development (61%), followed by respiratory (53%) and neurology (45%). Approximately one-quarter of all programs covered sleep-related cardiology (26%), ENT/otolaryngology (26%), and psychiatry/psychology (24%). Overall, the total time spent on sleep education across all sleep related domains ranged from a low of 50 minutes (Singapore) to a high of 788 minutes (India), with an average of 266 minutes across all programs, and significant differences across countries, *p* < .05. The median for each country ranged from 50 to 4200 minutes, with an average of 120 minutes. Overall, 23.3% of programs reported providing no sleep-related education and 94.8% of programs offer less than 10 hours of instruction. A small minority of programs (n = 6; 5.2%) offered over 10 hours of instruction and two of those programs (1.4%) report over 30 hours of education.

**Table 1:** Sleep education

**Country**	**HK**	**IN**	**ID**	**JP**	**KR**	**SG**	**TH**	**VN**	**US-CA**	**Total**
	**n = 3**	**n = 17**	**n = 9**	**n =35**	**n = 20**	**n = 2**	**n = 1**	**n = 1**	**n =64**	**n = 152**
**Specialty area (% of respondents reporting covering each topic)**	%	%	%	%	%	%	%	%	%	%
Cardiology	33	47	0	17	10	50	0	0	19	26
ENT/Otolaryngology	0	29	0	20	30	100	0	0	31	26
Neurology	33	53	0	54	60	100	0	0	39	45
Respiratory	100	71	11	43	60	100	100	100	55	53
Development	33	77	89	60	60	100	0	0	55	61
Psychiatry/Psychology	33	41	0	23	45	50	0	0	17	24
*Total (mean minutes)*	*420*	*788*	*77*	*420*	*281*	*50*	*180*	*120*	*162*	*266*
*Total (median minutes)*	*420*	*240*	*90*	*60*	*210*	*50*	*180*	*120*	*120*	*120*
**Specialty topics (% of respondents reporting covering each topic)**	%	%	%	%	%	%	%	%	%	%
Normal sleep	100	65	89	37	80	100	0	100	95	76
Delayed sleep phase disorder	67	12	44	17	30	50	0	0	58	38
Hypersomnia	100	41	33	20	55	50	0	0	73	52
Insomnia	100	35	33	23	45	50	0	100	59	45
Parasomnias	100	71	67	37	75	100	0	0	92	72
Behavioral insomnia of childhood	100	53	44	37	75	100	0	0	92	69
Sleep apnea	100	94	44	63	95	100	100	100	91	83
*Sleep related rhythmic movements*	*100*	*41*	*22*	*29*	*95*	*50*	*0*	*0*	*64*	*49*
*Sleep in medical disorders*	*67*	*41*	*22*	*23*	*25*	*100*	*100*	*0*	*39*	*33*

Note:

• Percentages presented are the percentage of respondents in each country that indicated a topic is covered in their curriculum.

• Zeros indicate that respondents reported no coverage of sleep education in general or for a specific topic.

Regarding sleep disorders, about 75% of programs covered normal sleep, parasomnias, behavioral insomnia of childhood, and sleep-related rhythmic movements. Delayed sleep phase disorder (38%) and sleep issues in medical disorders (33%) were covered the least. In some countries, such as Hong Kong, Singapore, and the United States, most programs covered most sleep domains. In other countries, such as Thailand and Vietnam, few domains were covered. Again, there were significant differences across countries for all disorders, p < .05.

### Non-didactic education

In addition to didactic education, non-didactic education was provided across a number of domains. The most common educational opportunities included journal club (53%), guest lectures (51%), grand round speakers (47%), and case consults (45%). Elective rotations were also common (40%), however non-elective rotations were less frequently provided (20%).

### Barriers to sleep education

The three most common barriers identified by the respondents, if sleep was not adequately covered in the curriculum, were lack of qualified staff (53%), insufficient time (47%), and a lower priority (46%). Other barriers included lack of resources (29%) and lack of relevance (9%).

## Discussion

In contrast to sleep-related education in medical schools, there appears to be more coverage of sleep and sleep disorders in pediatric residency programs, albeit limited. Overall, the median amount of time spent on sleep education was 2 hours (mean = 4.4 hours), with one-quarter of programs responding that their pediatric residency program provides no education regarding sleep. As expected, there were significant country-based differences, with one country providing very limited education (Indonesia), one country only focusing on sleep apnea/respiratory (Thailand), while other countries cover the breadth of sleep problems (Hong Kong, India, and United States-Canada). There was also significant differences in coverage based on topic area, with 83% of all programs indicating coverage of sleep apnea (and some countries reporting 100%), compared to just a third of programs covering such topics as delayed sleep phase syndrome and sleep in medical disorders.

Previously, a survey of U.S. medical schools conducted in 1990 reported that medical students received about 2 hours of sleep education, with 13% reporting no education in the preclinical years [6]. Similarly, a study of medical schools conducted in 2011 [7] found sleep education coverage of approximately 2 hours. In comparison, the mean hours of education in pediatric residency programs reported in 1994 in the US was 4.8 hours (median = 0). At that time, 46% of programs reported no inclusion of sleep education. Interestingly, the mean hours of education in this study was the exact same (although in the US it has actually decreased to 2.7 hours), although the median did increase substantially, as well as the percentage of programs that provide any education (77% vs 54%).

Interestingly, many programs report providing non-didactic educational opportunities, primarily through journal clubs, speakers, and case consults. Less than half have elective rotations, and only 20% offer a non-elective rotation. Compared to the previous study of 156 pediatric residency programs in the United States conducted in 1994 [8], this is a significant increase in the availability of non-elective (20% vs 4%) and elective rotations (40% vs 25%). All other non-didactic opportunities are either similar in prevalence or slightly less common than almost 20 years ago. Note that this study assessed education in multiple countries compared to just US programs from 1994.

The primary obstacles to providing sleep education were insufficient time and lack of qualified instructors. However, disappointingly, almost half of the programs indicated that the inclusion of pediatric sleep education was a low priority. Compared to the previous study of medical schools [7], this was a more prevalent barrier cited. Given that many of the residents in these programs will be involved in primary care, it is important to include education on how common pediatric sleep issues are a concern of parents, and that residents need to be educated on this frequent and important issue.

As with former surveys of this type, it is possible that the results provided overestimate the amount of sleep education provided in residency programs, as it is probable that those who responded were more likely to be from programs in which sleep education was provided. Excluding Japan, the response rate was higher than in previous studies, but still these results may not be representative of sleep education as a whole in residency programs. In contrast, the hours of education are probably an underestimate given that sleep concerns are likely to arise in daily patient care, both in primary care settings and in specialty clinics. Thus, sleep-related information that is imparted within these kinds of settings is unlikely to be reported. Furthermore, the exact hours of education reported was likely subject to interpretation by the program directors. That is, some program directors may have only considered didactic lectures in their reporting, whereas others may have included non-didactic teaching. Thus, the large discrepancies between countries, especially of those of similar level of development and educational maturity, may be due to differences in defining educational hours by the program director responding to the survey. Furthermore, future research should specifically survey pediatric residents about their perspective regarding sleep education in their residency programs.

Over the past years, there has been a significant increase in the recognition of sleep disorders in general, and specifically in pediatrics. In 2002, the American Academy of Pediatrics released guidelines that every child should be screened for snoring [11] and the in 2006 the American Academy of Sleep Medicine developed standards of practice guidelines for behavioral treatments of bedtime problems and night wakings [12]. Furthermore, practice parameters have been released giving indications for polysomnography in pediatric patients. However, in contrast, there appears to be a limited increase in sleep education provided in pediatric residency programs. Thus, there needs to be a continued push for inclusion of sleep education in the training of pediatric specialists.

## Conclusions

In contrast to sleep-related education in medical schools, there appears to be more coverage of sleep and sleep disorders in pediatric residency programs, albeit quite limited. These results indicate that there is still a need for more efforts to include sleep-related education in all pediatric residency programs, as well as coverage of the breadth of sleep-related topics. Such education would be consistent with the increased recognition of the importance of sleep and under-diagnosis of sleep disorders in children and adolescents.

## Competing interests

The authors declare that they have no competing interests.

## Authors’ contributions

All authors substantially contributed to the design of the study and data acquisition. JM performed the statistical analyses and drafted the manuscript. AB and DG revised the manuscript for intellectual content. All authors read and approved the final manuscript.

## Supplementary Material

Additional file 1: Pediatric Residency Sleep Education Survey. Click here for file
