# Supplementary material for: Sleep education in pediatric residency programs: a cross-cultural look
Source: BMC Res Notes. 2013 Apr 3;6:130. doi: 10.1186/1756-0500-6-130 (PMC3621514; doi:10.1186/1756-0500-6-130)
Supplement: Additional file 1: — Pediatric Residency Sleep Education Survey. [file 1756-0500-6-130-S1.doc]

**Appendix A**

**Pediatric Residency** Sleep Education Survey

| 1. **How much time (in hours and minutes) is allocated to formal didactic sleep education in the following areas during Paediatric Residency or Postgraduate Training Programs?** | | |  |
| --- | --- | --- | --- |
|  |  |  |  |
| |  | **Curriculum Area** | **Do you cover this area?** | | **If yes, please specify the amount of time** | | --- | --- | --- | --- | --- | |  | **YES** | **NO** | | 1 | Cardiology |  |  | 00hours 00 minutes | | 2 | ENT/Otolaryngology |  |  | 00hours 00 minutes | | 3 | Neurology |  |  | 00hours 00 minutes | | 4 | Respiratory/Pulmonology |  |  | 00hours 00 minutes | | 5 | Development |  |  | 00hours 00 minutes | | 6 | Psychiatry/Psychology |  |  | 00hours 00 minutes | | 7 | Other |  |  | 00hours 00 minutes | | | | |
|  |  |  |  |
|  | | |  |
| 1. **What topics of ‘sleep’ are covered in Paediatric Residency or Postgraduate Training Programs? (Please tick all that apply)** | | |  |
| |  | **Topics Covered** | **Do you cover this topic?** | | **If yes, please specify the amount of time** | | --- | --- | --- | --- | --- | |  | **YES** | **NO** | | 1 | Normal Sleep |  |  | 00hours 00 minutes | | 2 | Delayed Sleep Phase |  |  | 00hours 00 minutes | | 3 | Hypersomnia (e.g., narcolepsy) |  |  | 00hours 00 minutes | | 4 | Insomnia |  |  | 00hours 00 minutes | | 5 | Behavioural Insomnia of Childhood (bedtime problems and night wakings) |  |  | 00hours 00 minutes | | 6 | Parasomnias (e.g., sleep walking, sleep terrors) |  |  | 00hours 00 minutes | | 7 | Sleep Apnoea |  |  | 00hours 00 minutes | | 8 | Sleep Related Movement Disorders (e.g., restless legs syndrome, periodic limb movement disorder) |  |  | 00hours 00 minutes | | 9 | Sleep In Medical Disorders |  |  | 00hours 00 minutes | | | |  |

|  | | |
| --- | --- | --- |
|  |  |  |
| 1. **Do you offer alternative types of non-didactic instruction in your Paediatric Residency or Postgraduate Training Program? Please tick all that apply.** | | |
|  |  |  |
| |  | **Format** | **Please tick** | | --- | --- | --- | | 1 | Non-elective rotation |  | | 2 | Elective rotation |  | | 3 | Grand rounds speaker |  | | 4 | Guest lectures |  | | 5 | Case consultations |  | | 6 | Journal clubs |  | | | |
|  |  |  |
|  | | |
| **4. If sleep is not adequately covered in your curriculum, please tick all that apply.** | | |
| |  | Reason | **Please tick** | | --- | --- | --- | | 1 | Insufficient time in curriculum |  | | 2 | Lack of trained staff/qualified instructor |  | | 3 | Lack of resources |  | | 4 | Low priority |  | | 5 | Not relevant to the programme |  | | 6 | Other (please specify) |  | | | |
| 1. **Any other comments on Sleep Education in Paediatric Residency or Postgraduate Training Programs would be appreciated. Please fill in the space provided or on a separate page.** | | |
